# [^13^C]‐galactose breath test in a patient with galactokinase deficiency and spastic diparesis

**DOI:** 10.1002/jmd2.12205

**Published:** 2021-02-03

**Authors:** Can Ficicioglu, Didem Demirbas, Britt Derks, G. Shashidhar Pai, David J. Timson, Maria Estela Rubio‐Gozalbo, Gerard T. Berry

**Affiliations:** ^1^ Department of Pediatrics, Section of Biochemical Genetics The Children's Hospital of Philadelphia, University of Pennsylvania School of Medicine Philadelphia Pennsylvania USA; ^2^ Division of Genetics and Genomics, The Manton Center for Orphan Disease Research Boston Children's Hospital, Harvard Medical School Boston Massachusetts USA; ^3^ Department of Pediatrics Maastricht University Medical Centre Maastricht The Netherlands; ^4^ Department of Clinical Genetics Maastricht University Medical Centre Maastricht The Netherlands; ^5^ Medical University of South Carolina Children's Health, Division of Genetics Charleston South Carolina USA; ^6^ School of Pharmacy and Biomolecular Sciences University of Brighton Brighton UK

**Keywords:** galactosemia, GALK1 deficiency, in vivo galactose oxidation, stable‐isotope labeled galactose

## Abstract

Galactokinase deficiency is an inborn error of carbohydrate metabolism due to a block in the formation of galactose‐1‐phosphate from galactose. Although the association of galactokinase deficiency with formation of cataracts is well established, the extent of the clinical phenotype is still under investigation. We describe a 6‐year‐old female who was diagnosed with galactokinase deficiency due to cataract formation when she was 10 months of age and initially started on galactose‐restricted diet at that time for 5 months. She developed gait abnormality at 4 years of age. Breath tests via measurement of ^13^C isotope in exhaled carbon dioxide following ^13^C‐labeled galactose administration at carbon‐1 and carbon‐2 positions revealed oxidation rates within the normal range. The results in this patient strikingly contrast with the results of another patient with GALK1 deficiency that underwent breath testing with [1‐^14^C]‐galactose and [2‐^14^C]‐galactose. Extension of in vivo breath tests to other galactokinase patients is needed to better understand the pathophysiology of this disease.

**Synopsis**

Galactokinase deficiency is a rare hereditary galactosemia associated with cataracts; a normal breath test using ^13^C‐galactose was observed in the case patient despite formation of cataracts, indicating the need to better delineate the biochemical phenotype in patients with this very rare orphan disease.

## 1 INTRODUCTION

Galactosemia is caused by any defect in the Leloir pathway of galactose metabolism, which is comprised of galactokinase (GALK1), galactose‐1‐phosphate uridylyltransferase (GALT), UDP‐galactose 4′‐epimerase (GALE) enzymatic components, as well as the new type IV galactosemia due to galactose mutarotase (GALM).^1^ In our bodies, following lactose ingestion, the majority of the galactose in the blood is cleared by the liver through the Leloir pathway and converted to glucose, which is then exported and eventually broken down to carbon dioxide and water.

Galactokinase deficiency (OMIM #230200; type II galactosemia) is one of the hereditary galactosemias with an estimated prevalence <1:100.000, although this is likely to be higher for regions with a founder effect. This condition is considered to be mainly associated with cataracts, which resolve with galactose restriction. However, reports of patients with pseudotumor cerebri, hepatosplenomegaly, intellectual disability, recurrent seizures and deterioration of neurological function have been described.^2^, ^3^, ^4^ Hitherto, it is not clear whether severely decreased galactokinase (GALK1; EC 2.7.1.6) activity is the sole cause of a more severe phenotype.

Human galactokinase is a monomeric 42 kDa enzyme which catalyzes the ATP‐dependent phosphorylation of galactose and some structurally related monosaccharides.^5^, ^6^, ^7^, ^8^ It is a member of the galactokinase, homoserine kinase, mevalonate kinase, and phosphomevalonate kinase (GHMP) family of small molecule kinases.^9^ Like other members of this family, its structure comprises two domains arranged in a V‐shape with the active site at the base of this cleft.^10^ Disease‐associated variants are distributed throughout the structure.^7^, ^8^, ^11^, ^12^

Breath tests via measurement of carbon isotopes in exhaled carbon dioxide following intravenous or oral administration of a tracer compound labeled with a carbon isotope have been successfully used to study substrate oxidation in vivo for decades. This methodology has been repeatedly used to evaluate the whole‐body galactose oxidative capacity in patients with the more common type of hereditary galactosemia, galactose‐1‐phosphate uridylyltransferase deficiency, to establish the severity of the deficiency. It has only been performed once before in a patient with galactokinase deficiency. We report a patient who suffered galactokinase deficiency‐associated cataracts and yet revealed normal galactose breath test.

## 2 METHODS

Whole‐body galactose oxidation breath test was performed as previously described.^13^ After an overnight fast, the patient was administered an intravenous bolus of 100 mg [1‐^13^C]‐galactose or [2‐^13^C]‐galactose. Breath was collected at baseline and at 30, 60, 90, 120, 180, 240, and 300 minutes for measurement of ^13^C enrichment in CO_2_ in expired air as previously reported for patients with galactose‐1‐phosphate uridylyltransferase (GALT) deficiency.

## 3 RESULTS

### 3.1 Index case

The patient is a 6‐year‐old female, Asian/Pacific Islander with galactokinase deficiency who was referred for evaluation of a gait abnormality at 4 years of age, which developed following cataract surgery at 1 year of age and re‐institution of a normal diet. At 10 months of age, bilateral cataracts were recognized. Ophthalmological examination was compatible with typical “sugar cataracts,” characterized by central lens alterations due to the abundant presence of aldose reductase in the lens epithelial cells.^14^, ^15^ High levels of galactose are reduced to galactitol leading to an apparent osmotic phenomenon with cataract as a result. A lactose‐restricted diet was initially started as an erythrocyte galactokinase enzyme analysis revealed 3.3% residual activity (GALK activity 0.8 U/Hb; mean 24.3 U/Hb; range 14‐28 U/Hb) and the GALT activity was normal (GALT activity 23.8 U/Hb; control 19 U/Hb). Dietary lactose restriction was stopped after cataract surgery, at 15 months of age. The child enjoyed ice cream, cheese, and cow's milk.

At approximately 3 years of age, mother noted that her gait was unstable. Neurological evaluation revealed weakness in the lower extremities, 3+ patellar deep tendon reflexes and bilateral ankle clonus. An MRI at 4 years of age showed mild cortical atrophy. An EMG and nerve conduction studies were normal. The only other concern was short stature. A plasma T4 and growth hormone levels were normal as was a growth hormone stimulation test. A chromosome karyotype analysis was normal. The bone age was delayed (2 years and 6 months at chronological age of 4 years 6 months) compatible with constitutional delay of growth and puberty.

She was referred for evaluation. At 5 years of age, while on an unrestricted diet, the urine galactitol levels were 1066 μmol/mmol creatinine (normal: 12.7 ± 11.9; n = 19) and the erythrocyte galactose‐1‐phosphate level was 1.6 mg/dL (normal <1.0 mg/dL). The erythrocyte UDPglucose, UDPgalactose, and UDPglu/UDPgal ratio were 10.8 mg,/dL, 3.9 mg/dL, and 2.8, respectively. The normal values for erythrocyte UDPglucose, UDPgalactose, and UDPglu/UDPgal, respectively, are as follows: 10.2 ± 1.6, 4.5 ± 1.2, and 2.4 ± 0.5^16^ A repeat analysis revealed the erythrocyte UDPglucose, UDPgalactose, and UDPglu/UDPgal ratio as 17.5 mg/dL, 5.4 mg/dL, 3.3, respectively, as well as urine galactitol level of 450 μmol/mmol creatinine and galactose‐1‐phosphate level of <1.0 mg/dL.

On physical examination at 6 years of age, the patient was short and had an ataxic gait. The weight was 12.7 kg and height was 99.5 cm (mother's height: 150 cm; father's height: 167 cm). There was bilateral iridiodyskinesis. Neurologic findings were restricted to weakness of the lower extremities, bilateral hyperreflexia and sustained ankle clonus. Specific cerebellar findings including abnormal finger‐to‐nose test and abnormal speech were absent. A galactose‐restricted diet was reinstituted.

### 3.2 Additional investigations

**FIGURE 1:**
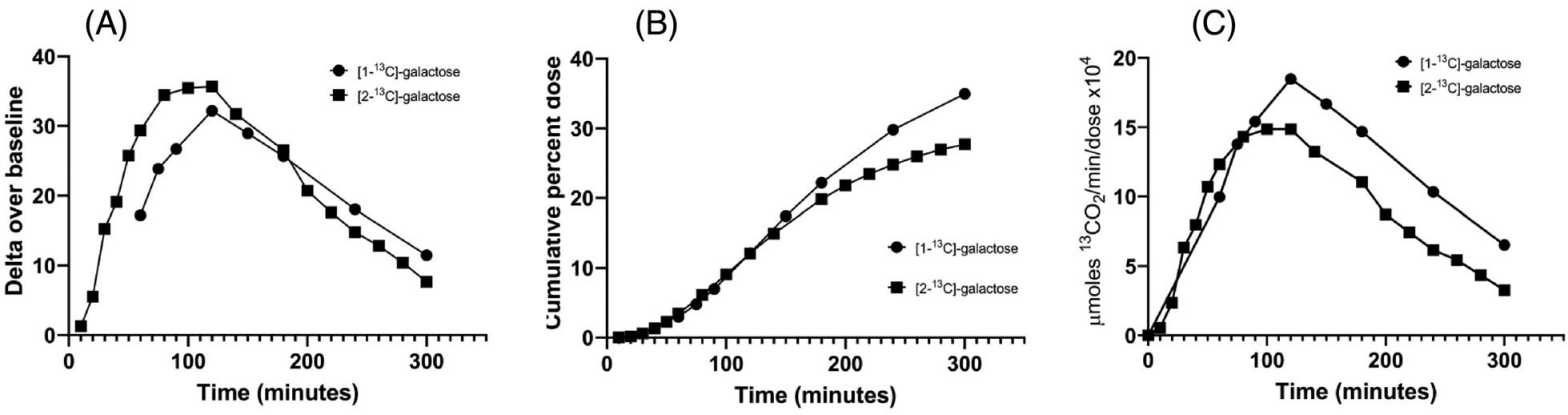
Breath tests results in the galactokinase deficiency patient after administration of 100 mg [1‐^13^C]‐galactose or [2‐^13^C]‐galactose. A, The difference between the ratio ^13^CO_2_/^12^CO_2_ in the expired air after administration of the dose is given as delta over baseline. B, Cumulative percent dose of ^13^CO_2_ over time is shown. C, Fractional elimination of the dose as the μmol ^13^CO_2_/min/μmol dose × 10^4^ over 5 hours is shown

Whole‐body galactose oxidation breath test revealed that the patient had eliminated 35% of the bolus [1‐^13^C]‐galactose as ^13^CO_2_ by 5 hours, which is within the normal range (Table 1, Figure 1). The breath test with [2‐^13^C]‐galactose was later performed and the fractional elimination as cumulative percent of the dose (CUMPCD) was 28%.

**TABLE 1:** Percent ^13^CO_2_ excretion in 5 hours using [1‐^13^C]‐galactose or [2‐^13^C]‐galactose

	Age	Gender	^13^C label position	Percent load excreted in 5 h
GALK patient [*index case*]	5	F	C1	35
			C2	28
GALT patient 1 (p.S135L/p.S135L)^a^	12	F	C1	18.9
			C2	26.2
GALT patient 2 (p.Q188R/p.Q188R)^a^	7	F	C1	3.6
			C2	4.9
GALT carrier 1 (p.S135L/Normal)	37	F	C1	25 (34% at 8 h)
			C2	31 (41% at 8 h)
GALT carrier 2 (p.Q188R/Normal)	35	F	C1	28 (40% at 8 h)
			C2	31 (44% at 8 h)
Control (Normal/Normal) Reference controls (n = 16)^a^	6‐56	9 F, 7 M	C1	Mean ± SD: 40.58 ± 7.65 Range: 27.26‐53.50^b^
Control (Normal/Normal)	11	F	C2	27

^a^ p.S135L: p.Ser135Leu; p.Q188R: p.Gln188Arg.

^b^ Reference^13^, Pediatric Research.

**FIGURE 2:**
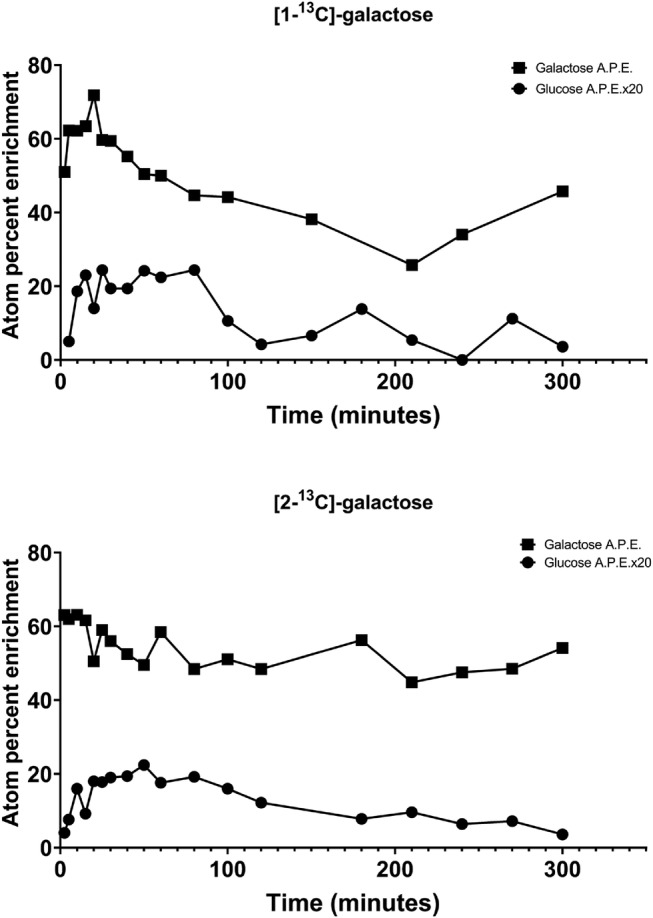
^13^C‐labeled plasma galactose and enrichment of ^13^C in plasma glucose after administration of [1‐^13^C]‐galactose (top panel) or [2‐^13^C]‐galactose (bottom panel) over time are shown

We also studied isotopically labeled galactose and glucose in these same studies to monitor the time‐dependent conversion of labeled galactose to labeled glucose (Figure 2). There is significant conversion of labeled galactose to labeled glucose in 5 hours with both [1‐^13^C]‐galactose and [2‐^13^C]‐galactose.

## 4 DISCUSSION

A case of galactokinase deficiency diagnosed at 10 months of age after manifestation of the classical sugar cataracts, which were then surgically removed, is presented. Dairy products were reintroduced in her diet at 15 months of age. The appearance of neurological problems at 3 years of age prompted us to perform additional investigations. in vivo testing studies with isotopically labeled galactose to delineate whole body galactose oxidation was performed for the first time in a galactokinase patient using ^13^C labeled galactose at two different carbon positions: [1‐^13^C] and [2‐^13^C]. To our knowledge, the only other study that investigated the whole‐body galactose metabolism of a galactokinase deficiency patient with breath testing was performed in 1974 using ^14^C labeled galactose, also using labels at carbon‐1 and carbon‐2 positions.^17^ Unlike the results of the 1974 study performed on the first patient described to have galactokinase deficiency, the results from our case were surprisingly normal. Despite deficient galactokinase activity in erythrocytes, whole body galactose handling was uncompromised.

Early in vivo studies on patients with GALT deficiency using [1‐^14^C]‐galactose demonstrated that individuals with classic galactosemia oxidize up to 8% galactose in 5 hours, whereas in control individuals, 30% to 35% of galactose is oxidized in that duration.^18^ Extensive in vivo studies using [1‐^13^C]‐galactose demonstrated that individuals with GALT deficiency oxidized an average of 4% of a given dose in 5 hours (compared to around 40% in controls) and eliminated approximately 17% to 50% of a 7 mg/kg bolus dose in 24 hours.^19^, ^20^, ^21^, ^22^, ^23^ Steady‐state studies with continuous infusion of labeled galactose suggest endogenous production of galactose in gram quantities per day even in classic galactosemia patients,^21^, ^22^ and this amount is estimated to be similar in galactokinase patients.^24^ As estimated urinary excretion of galactitol and galactonate account for only approximately 30% of daily burden of the endogenously made galactose in these galactosemic patients and to maintain steady‐state levels, further oxidation through non‐GALT pathways such as UDP‐glucose pyrophosphorylase are thought to be involved.^23^ These studies also showed that it is possible to distinguish between the severe and variant *GALT* genotypes through breath testing.^13^ This is especially helpful when the patient has a rare and previously unstudied mutation or manifest biochemical perturbations that are atypical in nature. Individuals with variant disease with substantial residual GALT activities demonstrated oxidative capacity comparable to controls. One striking example is the normal breath test shown in patients with the homozygous GALT p.Ser135Leu (c.404C > T) mutation.^13^, ^18^, ^20^, ^25^

In vivo studies to understand the utilization of galactose in individuals with GALT deficiency using labeled galactose is mostly performed with galactose labeled at C1 position. One early study used ^14^C‐galactose labeled at C1 or C2 positions over a 8 to 10 hours period.^25^ Results showed that approximately 8% of the [1‐^14^C]‐galactose was oxidized during this period while 4% of [2‐^14^C]‐galactose was oxidized into labeled carbon dioxide for individuals with GALT deficiency while for control individuals the oxidation rates of carbon‐1 or carbon‐2 labeled galactose were not significantly different. This prompted the researchers to postulate that the slow oxidation of galactose in galactosemic patients occurred via a directed oxidative pathway involving conversion of galactose to galactonate, which is subsequently decarboxylated to form d‐xylulose and can enter pentose phosphate metabolic pathway.

The data presented here on control individuals using [1‐^13^C]‐galactose and [2‐^13^C]‐galactose are similar to the earlier findings of Segal and Cuatrecasas using [1‐^14^C]‐galactose and [2‐^14^C]‐galactose.^25^ Furthermore, the data with [1‐^13^C]‐galactose on carriers of GALT deficiency and patients with severe classic galactosemia due to GALT deficiency as well as patients with hypomorphic forms due to p.Ser135Leu variant are comparable to our previous reports.^13^, ^20^, ^21^, ^22^ The percentage of the galactose load that appeared as labeled carbon dioxide in expired air in our case patient at 5 hours was 35% and 28% for [1‐^13^C]‐galactose and [2‐^13^C]‐galactose, respectively. These values are within the normal range as established by using [1‐^13^C]‐galactose measurements.^13^ The rate of excretion via the cumulative percent dose over time was very similar for both labels until approximately 2 hours. The carbon‐1 labeled galactose showed higher excretion rate thereafter (Figure 1). The results in this patient strikingly contrast the results of the other described patient with GALK1 deficiency that underwent breath testing with [1‐^14^C]‐galactose and [2‐^14^C]‐galactose. In that patient, low levels of ^14^CO_2_ production were measured comparable to that observed in patients with GALT deficiency.^17^ Unlike classic GALT deficiency with 0 % residual enzyme activity, where there is little labeling of glucose, the patient shows a significant amount of conversion in 5 hours, which is not surprising given the normal breath test results. The normal breath test in our case subject with galactokinase deficiency is not unlike with what you would see for an African American patient who is homozygous for the GALT p.S135L mutation ([p.Ser135Leu/p.Ser135Leu]), a disease in which significant pathology can develop in infancy with no treatment yet the galactose breath test with labeled galactose is normal.

Since the galactokinase deficiency leads to a blockade in the conversion of galactose to galactose‐1‐phosphate, subsequently less glucose‐1‐phosphate is available to enter the carbohydrate metabolism, initiating a decreased production of CO_2_. Tedesco et al.^26^ describe the possibility of a Philadelphia variant of the galactokinase gene, which mimics the phenotype of those heterozygous for galactokinase mutation and is mostly common in Black populations. Individuals with the Philadelphia variant show normal galactokinase activity in white blood cells and decreased galactokinase activity in red blood cells.^26^, ^27^ It is possible that this phenomenon in this patient is analogous to a patient with the African‐American mutation p.Ser135Leu in the *GALT* gene, for whom the residual enzyme activity in other organs is higher and enables a normal galactose oxidation. It is unclear whether the neurological abnormality is due to GALK1 deficiency. The cause of the upper motor neuron disease is still unknown and since the patient is lost to follow‐up, additional investigations are not possible. One possibility is that the patient had developed pseudotumor cerebri during the period when she was on an unrestricted diet.^28^ Theoretically, the brain edema may have impacted the corticospinal tracts. Lastly, as the normal breath testing demonstrated by our case patient is perplexing, extension of the in vivo breath testing to other galactokinase patients is needed to better understand the pathophysiology of this disease and the intricacies of galactose metabolism in humans.

## CONFLICT OF INTEREST

The authors have nothing to declare.

## AUTHORS CONTRIBUTIONS

Can Ficicioglu: Investigation, Resources, Writing – review and editing. Didem Demirbas: Conceptualization, Investigation, Writing – original draft, review and editing, Visualization. Britt Derks: Conceptualization, Investigation, Writing‐ original draft, review and editing, Visualization. G. Shashidhar Pai: Investigation, Patient Care, Resources, Writing – review and editing. David J. Timson.: Conceptualization, Investigation, Writing – original draft, review and editing, Visualization. Maria Estela Rubio‐Gozalbo: Conceptualization, Investigation, Writing‐ original draft, review and editing, Visualization. Gerard T. Berry: Conceptualization, Patient Care, Methodology, Investigation, Resources, Writing – original draft, review and editing, Visualization, Supervision.

## ETHICS STATEMENT

This study was approved by the IRB of The Children's Hospital of Philadelphia and mother provided informed consent.

**Abbreviations**
CUMPCD: cumulative percent of the dose
GALK1: galactokinase 1
GALT: galactose‐1‐phosphate uridylyltransferase
GALE: UDP‐galactose 4′‐epimerase
